# Focal Therapy for Prostate Cancer: Recent Advances and Insights

**DOI:** 10.3390/curroncol32010015

**Published:** 2024-12-28

**Authors:** Francesco Lasorsa, Arianna Biasatti, Angelo Orsini, Gabriele Bignante, Gabriana M. Farah, Savio Domenico Pandolfo, Luca Lambertini, Deepika Reddy, Rocco Damiano, Pasquale Ditonno, Giuseppe Lucarelli, Riccardo Autorino, Srinivas Vourganti

**Affiliations:** 1 Department of Urology, Rush University, Chicago, IL 60612, USA; francesco-lasorsa96@libero.it (F.L.); arianna_biasatti@rush.edu (A.B.); orsini.ang@gmail.com (A.O.); gabrielebignante@gmail.com (G.B.);; 2 Department of Precision and Regenerative Medicine and Ionian Area-Urology, Andrology and Kidney Transplantation Unit, University of Bari “Aldo Moro”, 70121 Bari, Italygiuseppe.lucarelli@uniba.it (G.L.); 3 Urologic Clinic, Department of Medicine, Surgery and Health Sciences, University of Trieste, 34127 Trieste, Italy; 4 Urology Unit, Department of Medical, Oral and Biotechnological Sciences, “G. d’Annunzio” University, 66013 Chieti, Italy; 5 Division of Urology, Department of Oncology, University of Turin, San Luigi Gonzaga Hospital, Orbassano, 10043 Turin, Italy; 6 Department of Urology, University of L’Aquila, 67100 L’Aquila, Italy; pandolfosavio@gmail.com; 7 Department of Neurosciences, Science of Reproduction and Odontostomatology, Federico II University, 80138 Naples, Italy; 8 Department of Experimental and Clinical Medicine, Careggi Hospital, University of Florence, 50121 Florence, Italy; 9 NHS Imperial College London, London W2 1NY, UK; 10 Department of Urology, Magna Graecia University, 88100 Catanzaro, Italy

**Keywords:** prostate cancer, focal therapy, ablation

## Abstract

Focal therapy has emerged as a balanced middle ground aiming to reduce overtreatment and the risk of progression, as well as patients’ distress and anxiety. Focal therapy and partial gland ablation prioritize the precise elimination of the index lesion and a surrounding safety margin to optimize treatment outcomes and lower the risk of residual disease. The paradigm of whole-gland ablation has shifted towards more targeted approaches. Several treatment templates ranging from subtotal and hemiablation to “hockey-stick”, quadrant, and even focal lesion ablation have emerged. Many types of energy may be utilized during focal treatment. First, focal therapy can be grossly classified into thermal vs. non-thermal energy. The aim of this non-systematic review is to offer a comprehensive analysis of recently available evidence on focal therapy for PCa.

## 1. Introduction

Focal therapy has emerged as a balanced middle ground aiming to reduce overtreatment and the risk of progression, as well as patients’ distress and anxiety [1,2,3].

Focal therapy and partial gland ablation prioritize the precise elimination of the index lesion and a surrounding safety margin to optimize treatment outcomes and lower the risk of residual disease [4]. Accurate multiparametric magnetic resonance imaging (mpMRI) planning is of utmost importance since the target lesions must be visible. Histological confirmation of the target lesions and the absence of clinically significant prostate cancer (csPCa ≥ ISUP2) on systematic biopsy are essential criteria for careful patient selection [5]. Table 1 presents the guidelines of the European Association of Urology (EAU) and the American Association of Urology/American Society for Radiation Oncology (AUA/ASTRO), and the recommendation of the FocAL therapy CONsensus (FALCON) [6,7,8].

**Figure 1:**
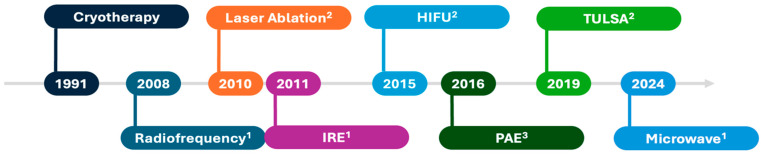
Timeline of Food and Drug Administration approval of focal therapy technologies: ^1^ approval for soft tissue ablation; ^2^ approval for prostate tissue ablation; ^3^ approval for benign prostatic hyperplasia treatment. IRE: irreversible electroporation; HIFU: high-intensity focused ultrasound; PAE: prostatic artery embolization; TULSA: transurethral ultrasound ablation.

Recently, the paradigm of whole-gland ablation has shifted towards more targeted approaches. Several treatment templates ranging from subtotal and hemiablation to “hockey-stick”, quadrant, and even focal lesion ablation have emerged [9]. Many types of energy may be utilized during focal treatment (Table 2). First, focal therapy can be grossly classified into thermal vs. non-thermal energy. Accordingly, the route of energy administration will differ, as well as the site of delivery and the anesthesia requirement [10]. Figure 1 depicts the timeline of the approval of the treatment energy by the Food and Drug Administration (FDA).

Expert consensus recommends a minimum follow-up period of 5 years for patients [11]. During the first year, prostate-specific antigen (PSA) levels should be evaluated every 3 months, with a prostate biopsy performed at the 1-year mark. The biopsy should include systematic sampling and 4–6 targeted cores from the treated area. Additionally, mpMRI should be repeated at 6 and 12 months following the initial imaging. Beyond the first year, PSA should be assessed every 6 months, and mpMRI should be performed annually [12].

The aim of this non-systematic review is to offer a comprehensive analysis of recently available evidence on focal therapy for PCa.

## 2. Literature Search

A literature search was conducted in September 2024 using the MEDLINE (via PubMed) and Embase databases by searching up-to-date publications. Articles not in English, not original investigations (such as editorials, commentaries, or abstracts), studies reporting experimental studies on animals or cadavers, and studies not reporting outcomes of focal therapy for PCa were excluded.

## 3. Thermal Energy

### 3.1. High-Intensity Focused Ultrasound (HIFU)

HIFU is a non-invasive, non-ionizing technique in which ultrasound waves (>500 W/cm^2^) are used to cause coagulative necrosis by transmission from the transducer. In this technique, an endocavity transrectal probe is used, without requiring tissue penetration. An endorectal balloon ensures rectal protection via water circulation during the procedure [13]. Zones of high and low pressures are formed in the tissue as ultrasound energy propagates and is converted into heat (>65 °C) [14]. Real-time diagnostic ultrasound (US) is used to guide the treatment during ablation. HIFU treatment can be distinguished as either algorithm-directed or visually directed [14]. In algorithm-directed HIFU, specific tissue properties, homogeneity, and fixed ultrasound absorption coefficients are assumed to produce thermal ablation using predefined power and time settings at designated tissue depths. Conversely, visually directed HIFU enables real-time visualization of prostate tissue changes, allowing the user to adjust power settings [15]. Transrectal HIFU devices approved by the Food and Drug Administration (FDA) are Sonablate (SonaCare Medical LLC, Charlotte, NC, USA), Ablatherm (EDAP TMS, Vaulx-en-Velin, France), and Focal-One (EDAP TMS, Vaulx-en-Velin, France) [16].

The Sonablate device positions the patients in the dorsal lithotomy position and is compatible with MR–US fusion technology. Intraprostatic cysts and calcifications may affect treatment, and the ideal total thickness (anterior–posterior prostate diameter and rectal wall) should be less than 37 mm, with focal lengths of 30 and 40 mm available for use [17]. For the Ablatherm device, the patient is secured in the right lateral decubitus position. Large cysts (>10 cc) within the treatment area represent a contraindication for its use. The prostate target dimension should have a maximum anteroposterior diameter of 24 mm. Volume reduction surgery (typically TURP) has been utilized in order to limit postoperative urinary symptoms, debulking larger glands for technically feasible treatment, and removing calcifications [18]. The Focal-One device is the successor to the Ablatherm and is recognized for its benefits, including MRI–TRUS fusion, real-time adjustments to treatment areas, and the capability to target depths up to 40 mm in diameter [10].

A recent systematic review provided the oncological and functional outcomes of whole-gland HIFU. The negative biopsy rates after HIFU were 20–92.7%, with 5-year biochemical disease-free survival ranging from 21.7% to 92%. The main postoperative complications were urinary retention, hematuria, urinary tract infection (UTI), rectourethral fistula, and retrograde ejaculation. The occurrence of urinary stress incontinence and de novo erectile dysfunction has also been described after whole-gland HIFU [19]. In contrast, HIFU partial-gland ablation has been demonstrated to improve functional outcomes and reduce complications compared to whole-gland approaches. This improved side effect profile can be achieved while maintaining oncologic outcomes in well-selected patients. At 1-year follow-up biopsy, 24.2% and 12.6% of subjects were diagnosed with any cancer and clinically significant cancer (grade group ≥ 2) after partial ablation, respectively [20]. One challenge to follow-up surveillance is that the diagnostic performance of MRI in detecting recurrence is imperfect in this setting due to its variable sensitivity and specificity, which prevents it from replacing biopsies [21].

### 3.2. Transurethral Ultrasound Ablation (TULSA)

TULSA utilizes continuous sweeping directional but non-focused ultrasound from an applicator in the prostate through the urethra (TULSA-PRO, Profound Medical Inc., Toronto, ON, Canada). The procedure is performed under real-time MRI thermometric guidance [22]. After treatment, the ablated volume appears on contrast-enhanced MRI as a non-perfused volume, indicating total cell death [23]. Targeting lesions in the anterior gland may be more effective with TULSA, as it permits an ablation zone of up to 3 cm surrounding the urethra. Similar to HIFU, contraindications include calcifications (>1 cm) and prostatic cysts within the treatment zone [10].

Klotz et al. first analyzed the outcomes of TULSA in 115 men [24]. No evidence of recurrence was detected in 65% of men at 12-month biopsy, while 75% of them maintained or regained erections. Globally, 7–17% of men receiving TULSA as a primary treatment for PCa underwent salvage treatment as per a systematic review. The preservation of potency and urinary continence ranged from 75% to 98% and 92% to 100%, respectively [25]. UTI (29–55%), hematuria (17–37%), and urinary retention (9–27.5%) were highlighted as postoperative complications [4].

### 3.3. Cryotherapy

Cryotherapy represents another well-established thermal focal therapy for PCa. Cryotherapy exerts its action by cycles of freezing tissues at the lethal temperature of −40 °C and subsequent thawing [26]. The patient is positioned in the dorsal lithotomy position, and cryoneedles are placed transperineally (1–1.5 cm apart) under the guidance of TRUS or MRI–US fusion [10]. At the lethal temperature, apoptosis occurs as a result of various pathophysiologic mechanisms such as cell dehydration with protein denaturation, direct rupture of membranes from ice crystals, and induction of microthrombi causing ischemic necrosis [27]. To limit urinary and sexual side effects reported after whole gland treatment, cryotherapy is now recommended for partial gland ablation [28]. Tumor size and shape, non-tumor tissue volume, and the surrounding structures are pivotal in choosing the number of needles. Indeed, the temperature of the ice ball that forms around the needle increases linearly away from it, reaching 0 °C at its edge, with a central 1 cm core that is −40 °C [28]. Real-time monitoring of the treatment is possible on US (areas of anechogenicity) supplemented with thermocouples for direct temperature monitoring [29].

A pooled rate of positive biopsy of 20.0% (95% CI 12.3–27.6%) was calculated in a recent meta-analysis, and biochemical recurrence-free survival was 75.7% (95% CI 67.3–84.2%) [30]. As with other modalities, the ability of MRI to identify recurrence post-ablation is imperfect. Wysock et al. addressed the ineffectiveness of mpMRI during the decision-making process for a 6-month biopsy [31]. The incidence of postoperative lower urinary tract symptoms has been reduced by the adoption of newer generation devices, the use of thermosensors at the level of the external urinary sphincter and rectal wall, and the use of a warming urethral catheter [32]. Overall, following treatment, the incidence of erectile dysfunction and total incontinence range from 58.1% to 90% and 0% to 3.6%, respectively [33].

### 3.4. Focal Laser Therapy (FLA)

Platforms utilizing laser energy have been developed for use directly within the MRI gantry. Coagulative necrosis is the result of the heat (photothermal effect) delivered by laser fibers either with a transrectal (prone position) or transperineal approach (supine position). In the latter case, an endorectal coil is used to stabilize the gland. Previous studies already confirmed the effectiveness of transperineal laser ablation (TPLA) for benign prostatic hyperplasia [34]. Upon identifying the area to be treated with MRI–US, a needle allows the advancement of an optical fiber [35]. MRI thermometric guidance may provide real-time monitoring of the treatment [10]. Laser ablation is suitable for all regions of the gland, though for posterior midline lesions, hydro-dissection should be considered to protect the rectum [36]. In-field and out-of-field recurrence were 23% and 7% in the largest series from Feller et al. [37]. Similar results were reported in a recent systematic review of in-field recurrence (7–20%) and csPCa-freedom rate (80–87.5%). Complications included UTI (0–4.1%), hematuria (8.2–100%), urinary retention (4.9–27%), and fistula (0–1.7%) [14]. Newer platforms utilizing MRI–US fusion have allowed such techniques to be delivered in more clinical settings outside of the MRI suite. Indeed, a multicenter pilot study pointed to the feasibility of TPLA under local perineal anesthesia [38].

## 4. Non-Thermal Energy

### 4.1. Irreversible Electroporation (IRE)

IRE utilizes high-voltage (up to 1500 V), low-energy electric pulses to create nanopores on prostate cells’ membranes, provoking their apoptosis. The short duration of the impulses (70 msec) minimizes any injuries to neurovascular bundles [39]. The targeted areas are preliminarily defined based on template-guided mapping prostate biopsies and mpMRI [40]. The patient is secured in the dorsal lithotomy position and has three to six (spaced 1–2 cm apart) parallel electrodes inserted transperineally while under general anesthesia with muscle relaxants [41]. The needle electrodes are subsequently attached to their corresponding cables in the Nanoknife system (AngioDynamics, Latham, NY, USA) [42]. When in close proximity to the rectum, a safety margin of at least 5–7 mm should be used to minimize the risk of fistula [10]. Ventricular arrhythmias, pacemakers, or defibrillation devices represent contraindications for IRE since the emission of pulses may induce arrhythmias. Moreover, it is crucial to avoid performing IRE when stents, surgical staples, or other metallic devices are close to the ablation area, as they can interfere with the electric field [42].

The pooled proportion of positive biopsies was 24.2% (95% CI 17.7–30.7%) after IRE [26,30]. A multicenter randomized study compared the results of focal and zonal IRE. No significant differences were noted in the recurrence of any-grade PCa (56.3 vs. 43.4%) and clinically significant PCa (18.7 vs. 13.2%) [43]. As for the imaging-based detection of recurrence, some studies have reported that MRI had a higher number of false positives compared to histologically confirmed cases [39]. After IRE, grade III–IV complications are rare. Similar to complications after transperineal prostate biopsy, more common events (grade I–II) are hematuria (5.6–24%), acute urinary retention (5.6–20%), UTI (9–21%), dysuria (15–26%), temporary urinary incontinence, and perineal pain [44].

### 4.2. Microwave Ablation

Microwave energy is delivered via needles placed transrectally or transperineally into target lesions under MRI–US fusion guidance to cause coagulative necrosis [45]. Barry Delongchamps et al. reported the results of the “first-in-human” trial of microwave ablation in 10 patients [46]. After proper measurements of the thermal effects in the three planes from the tip of the applicators, the transrectal approach was performed. This approach prevented the ablation of visible lesions in two patients: one with a large anterior lesion and another with a smaller lesion, whose largest axis was oriented transversely. The authors highlight that this failure was not related to the energy source. The transrectal approach better allows the ablation of lesions oriented perpendicularly.

The outcomes of the first phase 2 trial of the transperineal approach were investigated by Chiu et al. after treating 23 lesions in 15 patients [47]. At the 6-month biopsy, 2/23 and 5/15 in-field and out-of-field recurrences were proven, respectively. At the 6-month follow-up, no changes were reported in terms of urinary symptoms, uroflowmetry, erectile function, and overall quality of life.

### 4.3. Phototherapy

Photothermal and photodynamic therapies are the primary phototherapy strategies utilized for the treatment of PCa [48].

Photothermal therapy (PTT) is a minimally invasive treatment modality that relies on the activation of photothermal agents that are responsible for the conversion of near-infrared light energy into heat at the treatment site [49]. This leads to localized hyperthermia (>51 °C) and destruction of cancer cells. The wavelength, exposure time, and radiation intensity, as well as the characteristics and concentration of photothermal agents, are critical factors that influence the mechanisms of cell death [50]. Over time, numerous agents have been developed, including metal-based (such as gold nanoparticles), inorganic, and organic nanomaterials [49]. A clinical pilot study investigated nanoparticles with a silica core and a gold shell (AuroLase Therapy). Vessel fenestrations and defective lymphatic drainage facilitate the accumulation of these gold–silica nanoshells within tumor lesions. In the ablation zones, coagulative necrosis was confirmed, with 14 out of 16 biopsies indicating no residual tumor [51].

Vascular-targeted photodynamic therapy (PDT) requires photosensitizing agents to be administered intravenously and subsequently activated (photooxidation) using transperineal laser fibers [48]. These fibers emit light at a wavelength of 753 nm, with a fixed power density of 150 mW/cm for a duration of 22 min and 15 s [52]. Padeliporfirin is the most studied agent so far [53]. After its activation, superoxide and hydroxyl radicals are generated and cause vessel thrombosis, apoptosis, and necrosis, then non-thermal hemiablation of the gland [9].

The advantages of PDT include its independence from thermal energy and the absence of the heat sink effect [54]. The outcomes were first described by Azzouzi et al. when comparing PDT to active surveillance in men with low-risk, localized prostate cancer (Gleason 4 or 5 were excluded) [55]. After a median follow-up time of 24 months for the entire population, a significantly lower percentage of patients experienced disease progression after PDT (28 vs. 58%, *p* < 0.0001). Similarly, 7% and 24% of men underwent a radical treatment (radical prostatectomy or radiotherapy), compared to 32% and 53% in the active surveillance group after 2 and 4 years, respectively [56].

In terms of complications, hematuria and urge incontinence were found to be the most common ones, resolving within 6–8 weeks [57]. Overall, the pooled rate of positive biopsy was 36.2% (95% CI 28.6–43.8%) after PDT [30].

### 4.4. Radiofrequency Ablation

Transperineal needles deliver medium-frequency alternating current to generate heat and induce necrosis (Encage TM, Trod Medical, St Petersburg, FL, USA) [9]. Tissue impedance is monitored throughout the procedure. Recent series reported encouraging oncologic outcomes, with 70–80% of negative biopsy during follow-up [58,59,60]

## 5. Non-Ablative Approaches

### 5.1. Prostatic Artery Embolization

In 2017, the FDA approved prostatic artery embolization (PAE) for the treatment of LUTS secondary to benign prostatic hyperplasia (BPH) [61]. The femoral artery is accessed to evaluate the iliac and the prostatic arteries, usually under local anesthesia. Then, microsphere particles (300–500 μm) are injected to embolize the prostatic artery unilaterally. The infarcted areas exhibit specific patterns on post-procedure MRI: hyperintensity on T1WI and hypointensity on T2WI. However, they tend to become hypointense over time, while showing reduced signal intensity on DWI beginning one month after the intervention [62]. The most typical complications after PAE include the cystic transformation of the infarct area (hyperintense on T2WI and hypointense on T1WI) and “post-embolization syndrome” (pain, nausea, vomiting, fever, and dysuria) [62].

PAE was first described in an advanced-stage PCa or as a palliative treatment [63,64]. Later, Frandon et al. evaluated its feasibility in patients with low-risk disease with a single lesion on MRI [65]. Given the possibility of embolizing a whole gland lobe, PAE is possible for many prostate locations. The authors also discuss the limitations of mpMRI in the follow-up setting, noting that its findings may exhibit a delayed response when compared to biopsy results. Randomized and multicentric studies are warranted to confirm the promising results of PAE for PCa.

### 5.2. Partial Prostatectomy

Anterior lesions of the prostate, particularly those located at the apex, present significant challenges for focal ablative therapy [66]. The anterior location (transition zone and/or anterior fibromuscular stroma) increases the risk of thermal diffusion injury to critical structures such as the external striated sphincter, neurovascular bundles, and urethra, while also complicating treatment due to interference from the pubic symphysis [67]. Therefore, surgical excision of this region of the gland could preserve the posterior–lateral portions of the distal urethra, peripheral zone, and periprostatic tissues intact [68].

In 2016, Villers et al. demonstrated the feasibility of robot-assisted anterior partial prostatectomy in a highly selected cohort of 17 patients [67]. At 2 years, the recurrence-free survival was 0.86 (range: 0.55–0.96), and functional outcomes were encouraging. The adoption of a single-port robotic platform has led to the development of a transvesical approach that preserves the space of Retzius [66]. Notably, larger glands, calcifications, and obliterated prostatorectal junctions did not constitute contraindications for this technique, unlike with HIFU. The multifocal nature of many prostate cancers presents a major concern for partial prostatectomy.

### 5.3. Focal Brachytherapy

Genitourinary, gastrointestinal, and sexual side effects are typically associated with whole prostate irradiation [9]. Contrary to external beam radiation, transperineally placed implants deliver radiation into the targeted lesion, thus reducing the risk of side effects and enhancing patients’ recovery. Low dose rate (LDR) and high dose rate (HDR) represent the main approaches for focal brachytherapy [69]. LDR brachytherapy is a recognized treatment option for men with low to intermediate-risk PCa, while HDR brachytherapy is an emerging alternative, with studies indicating favorable oncological outcomes [70].

In the case of LDR brachytherapy, permanent implants administer a dose rate of less than 2 Gy per hour. Conversely, for HDR brachytherapy, higher doses (exceeding 12 Gy per hour) are administered through catheters, which are removed after the prescribed dose has been delivered [71]. Notably, HDR brachytherapy has been noted to have fewer common genitourinary adverse events than LDR brachytherapy [72]. The results of focal brachytherapy as a definitive treatment have been recently presented [73]. After a median follow-up of 4 years, the biochemical relapse-free survival was 0.91 (CI 95% 0.82–0.95). To limit the cost and invasiveness of brachytherapy, stereotactic body radiation therapy (SBRT) represents a strategy using external beams. Doses of 6.5–10 Gy are usually administered in five fractions [74]. Promising oncological outcomes have already been reported for SBRT, without an increase in acute or late toxicities [75].

## 6. Conclusions

Promising results have been shown so far by many different energy sources for focal therapy. Adverse events may occur even for non-thermal energy approaches (Supplementary Table S1). Effective patient selection and counseling are essential due to the considerable risk of oncologic failure, which includes both in-field and out-of-field recurrences. It is important to inform patients about the possibility of requiring further therapeutic interventions in the future. The absence of comparative trials involving different energy modalities continues to signify an unmet need.

## Figures and Tables

**Table 1:** EAU and AUA/ASTRO guidelines, and FALCON (FocAL therapy CONsensus) recommendation for focal therapy by disease stage. AS: active surveillance.

	EAU	AUA/ASTRO	FALCON
Low-risk disease	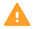 Trial setting or registry	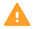 AS preferred	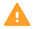 AS preferred
Intermediate-risk disease	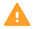 Trial setting or registry	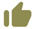 Select and inform patients	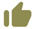 Recommended
High-risk localized disease	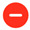 Not recommended	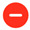 Not recommended	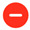 Not recommended
Locally advanced disease	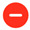 Not recommended	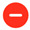 Not recommended	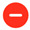 Not recommended

**Table 2:** Types of focal therapy for prostate cancer: NMB: neuromuscular blockade; HIFU: high-intensity focused ultrasound; TULSA: transurethral ultrasound ablation; IRE: irreversible electroporation.

	Type of Energy	Route of Delivery	Patient Positioning	MRI (in Gantry)	TRUS/MR Fusion	Sedation
HIFU	Thermal	Transrectal probe	Lithotomy/ right lateral decubitus		X	Anesthesia
TULSA	Thermal	Transurethral	Lithotomy	X		Anesthesia
Cryotherapy	Thermal	Transperineal needles	Lithotomy		X	Anesthesia or local
Laser therapy	Thermal	Transrectal or transperineal needles	Prone/supine	X	X	Anesthesia or local
IRE	Non-thermal	Transperineal electrodes	Lithotomy		X	Anesthesia (complete NMB)
Microwave ablation	Non-thermal	Transrectal or transperineal needles	Lithotomy		X	Anesthesia
Photodynamic therapy	Non-thermal	Intravenous photosensitizer + transperineal laser fibers	Lithotomy		X	Anesthesia
Radiofrequency ablation	Non-thermal	Transperineal needles	Lithotomy		X	Anesthesia

## Supplementary Materials

The following supporting information can be downloaded at: https://www.mdpi.com/article/10.3390/curroncol32010015/s1, Table S1. Reported outcomes and complications of focal therapy for prostate cancer.
