# Supplementary material for: Focal Therapy for Prostate Cancer: Recent Advances and Insights
Source: Curr Oncol. 2024 Dec 28;32(1):15. doi: 10.3390/curroncol32010015 (PMC11764426; doi:10.3390/curroncol32010015)
Supplement: Supplementary file 1 [file curroncol-32-00015-s001.zip › curroncol-3355294-supplementary.pdf]

Supplementary Table S1. Reported outcomes and complications of focal therapy for prostate cancer.
HIFU: high-intensity focused ultrasound; TULSA: transurethral ultrasound ablation; IRE: irreversible electroporation. N/R: not reported; UTI: urinary tract infection.
[1]: negative-biopsy rate; [2]: polled proportion of positive biopsy. [3]: clinically significant PCa-freedom rate.

	**Oncological Outcomes**	**Functional Outcomes**	**Complications**
HIFU	20-92.7%[1]	N/R	Urinary retention<br>UTI<br>Retrograde Ejaculation
TULSA	65%[1]	ED: 2-25%<br>Incontinence: 0-8%	UTI<br>Hematuria<br>Urinary retention
Cryotherapy	20.0% (12.3–27.6%)[2]	ED: 58.1- 90%<br>Incontinence: 0-3.6%	UTI<br>Urinary retention
Laser therapy	80-87.5%[3]	N/R	UTI<br>Hematuria<br>Urinary retention<br>Fistula
IRE	24.2% (17.7–30.7%)[2]	N/R	Hematuria<br>Urinary retention<br>UTI<br>Dysuria<br>Temporary urinary incontinence<br>Temporary perineal pain
Microwave ablation	8.7%[2]	N/R	N/R
Photodynamic therapy	36.2% (28.6–43.8%)[2]	N/R	Hematuria<br>Urge incontinence
Radiofrequency ablation	20-30%[2]	N/R	Hematuria<br>Hematospermia<br>Erectile dysfunction<br>Urethral stricture
